# Intraoperative detection and removal of microscopic residual sarcoma using wide-field imaging

**DOI:** 10.1002/cncr.27458

**Published:** 2012-03-21

**Authors:** Jeffrey K Mito, Jorge M Ferrer, Brian E Brigman, Chang-Lung Lee, Rebecca D Dodd, William C Eward, Lisa F Marshall, Kyle C Cuneo, Jessica E Carter, Shalini Ramasunder, Yongbaek Kim, W David Lee, Linda G Griffith, Moungi G Bawendi, David G Kirsch

**Affiliations:** 1 Department of Pharmacology and Cancer Biology, Duke University Medical CenterDurham, North Carolina; 2 Department of Chemistry, Massachusetts Institute of TechnologyCambridge, Massachusetts; 3 Department of Orthopedic Surgery, Duke University Medical CenterDurham, North Carolina; 4 Department of Radiation Oncology, Duke University Medical CenterDurham, North Carolina; 5 Department of Clinical Pathology, College of Veterinary MedicineSeoul National University, Seoul, South Korea; 6 David H. Koch Institute for Integrative Cancer Research, Massachusetts Institute of TechnologyCambridge, Massachusetts; 7 Department of Biological Engineering, Massachusetts Institute of TechnologyCambridge, Massachusetts

**Keywords:** optical molecular imaging, intraoperative imaging, soft tissue sarcoma, cathepsin proteases

## Abstract

**BACKGROUND:** The goal of limb-sparing surgery for a soft tissue sarcoma of the extremity is to remove all malignant cells while preserving limb function. After initial surgery, microscopic residual disease in the tumor bed will cause a local recurrence in approximately 33% of patients with sarcoma. To help identify these patients, the authors developed an in vivo imaging system to investigate the suitability of molecular imaging for intraoperative visualization. **METHODS:** A primary mouse model of soft tissue sarcoma and a wide field-of-view imaging device were used to investigate a series of exogenously administered, near-infrared (NIR) fluorescent probes activated by cathepsin proteases for real-time intraoperative imaging. **RESULTS:** The authors demonstrated that exogenously administered cathepsin-activated probes can be used for image-guided surgery to identify microscopic residual NIR fluorescence in the tumor beds of mice. The presence of residual NIR fluorescence was correlated with microscopic residual sarcoma and local recurrence. The removal of residual NIR fluorescence improved local control. **CONCLUSIONS:** The authors concluded that their technique has the potential to be used for intraoperative image-guided surgery to identify microscopic residual disease in patients with cancer. Cancer 2012. © 2012 American Cancer Society.

## INTRODUCTION

When surgeons resect a soft tissue sarcoma of the extremity, they attempt to preserve limb function while removing a margin of normal tissue surrounding the tumor so that no malignant cells remain. For soft tissue sarcomas, surgical margin status correlates with local recurrence, development of distant metastases, and disease-specific survival.^1^–^3^ The presence of tumor cells at the surgical margin of the resected specimen suggests that residual tumor cells may remain in the tumor bed. Margin status can be evaluated by a pathologist at the time of resection. However, during surgery, time constraints normally limit assessment to 1 or 2 small areas of the tumor, making this analysis prone to sampling error. The remaining excised tissue is fixed in formalin, and a more comprehensive assessment of the margin may take days to complete. If tumor cells are identified at the margin, then patients may require a repeat surgical resection,^4^ leading to increased patient morbidity and health care costs. Therefore, a method to directly assess the entire tumor bed at the time of surgery has the potential to improve therapy.

Intraoperative margin assessment in cancer patients has been accomplished using radiofrequency spectroscopy.^5^ Although initial studies with this technology have been encouraging, the limitations of this approach include an inability to resolve small clusters of tumor cells and an analysis of excised tissue rather than the surgical bed. An alternative approach for intraoperative tumor bed imaging is microscopy (eg, confocal). Microscopy in the operative setting requires scanning technology with rigid positioning of the detector relative to the tumor bed to maintain the focal distance. These restrictions can be cumbersome and too time-consuming to scan an entire tumor bed during surgery. More recently, others have proposed using exogenous, cancer-labeling, near infrared (NIR) imaging agents to guide surgical resection of tumor tissue.^6^

We have used optical imaging with epi-illumination during surgery to identify residual cancer with millimeter resolution in mice with primary soft tissue sarcomas.^7^ Although this approach has potential drawbacks, including limited tissue penetration,^8^ so that signal deep to the exposed tumor bed may not be detected, we hypothesized that NIR fluorescence with epi-illumination could be used to detect microscopic residual disease in a tumor bed after the surgeon removes all grossly apparent sarcoma.

To test this hypothesis, we used genetically engineered mice with conditional mutations in *B-raf* and *p53* that develop primary sarcomas. In contrast to xenograft models, this primary model has an intact immune system that more closely resembles the tumor microenvironment in human patients. Furthermore, these tumors frequently have poorly defined capsules and are highly invasive into the adjacent normal muscle.^7^ After intravenous injection of NIR fluorescent probes, we performed surgery guided by intraoperative imaging using a wide field-of-view imaging device capable of resolving microscopic clusters of tumor cells. Here, we tested the ability of optical imaging with epi-illumination to detect microscopic residual sarcoma during surgery.

## MATERIALS AND METHODS

### Microarray Analysis of Cathepsin Expression

Microarray data were downloaded from Gene Expression Omnibus (GEO) (GSE16779) and normalized as described previously.^9^ The mean expression of cathepsin proteases between tumor and normal muscle was identified using the statistical package BRB-arraytools (available from: http://linus.nci.nih.gov/BRB-ArrayTools.html [Access May 2011]).

### Mice and Sarcoma Generation

All mouse work was performed in accordance with Duke University Institutional Animal Care and Use Committee-approved protocols. The mouse genotypes that were used to generate sarcomas included *LSL-Kras^G12D/+^*; *p53^Flox/Flox^*^7^, *LSL-YFP*;*LSL-Kras^G12D/+^*;*p53^Flox/Flox10^*, *Braf^Ca/+^; p53^Flox/Flox^*, and *Braf^Ca/Ca^;p53^Flox/Flox^*^11^. The *LSL-YFP* mice^10^ were obtained from Jackson Laboratory (Bar Harbor, Me). Soft tissue sarcomas were generated in the proximal portion of the medial or lateral gastrocnemius muscle as previously described.^7^

### Imaging Device

Fluorescence excitation illumination was provided by a 300-Watt Xenon lamp (Sunoptic Technologies, Jacksonville, Fla.) and was transmitted into the device through an optical fiber bundle (Sunoptic Technologies). An achromatic doublet lens (Thorlabs, Newton, NJ) is used to collimate the fiber bundle light output. Input light is reflected by a cube-mounted mirror (Thorlabs) toward a band-pass excitation filter to limit the illumination to a narrow band that matches the absorption spectrum of the fluorophore in use. The filtered excitation light is reflected toward the specimen by a dichromatic mirror. A lens pair sends collimated illumination into the specimen plane and collects the fluorescence emission. After passing through the dichromatic mirror, the emission light is filtered by a band-pass optical element. The image is relayed onto a charge coupled device (CCD) (PixelFly QE; PCO AG, Kelheim, Germany) by an achromatic doublet lens (Thorlabs). The CCD camera is connected to a computer for image acquisition and display. Data acquisition software was written in LabView (National Instruments, Austin, Tex). Image analysis was performed using MatLAB (Mathworks, Natick, Mass) and ImageJ (National Institutes of Health, Bethesda, Md).

### Device Characterization

To compare 2 raw images with different exposure times, we adopted a previously described approach.^12^ Raw pixel counts from each image are normalized by exposure time to obtain a time-independent parameter with units of pixel counts per second. For exposure time calibrations, 15-μm fluorescent microspheres (Life Technologies, Grand Island, NY) were introduced into a flow channel and imaged at different exposure times ranging from 1 to 250 msec. The fluorescence intensity of 20 microspheres was measured at each exposure time.

To calibrate for intensity levels, 6-μm fluorescent microspheres with various fluorescence emission levels (Life Technologies) were imaged in a flow channel using the device. The nominal fluorescence ranged from 0.4% to 100% (normalized by the brightest microspheres). The fluorescence emission of 15 microspheres was measured for each nominal relative fluorescence. The average and standard deviation of the fluorescence emission in a given image was calculated and plotted against the manufacturer's nominal relative intensity.

The spatial resolution of the device was determined by imaging a US Air Force 1951 standard calibration target. The highest and minimum intensities (I_max_ and I_min_, respectively) of 2 consecutive lines for several cycles/mm groups were determined by image analysis using ImageJ. The modulus of the contrast transfer function (CTF) was determined as follows:

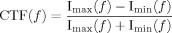

where *f* is the spatial frequency in line cycles/mm.

### Quantifying the Tumor-to-Muscle Signal Fluorescence Intensity Ratio

Mice with primary soft tissue sarcomas were injected with 2 nmol of Prosense 680, Prosense 750, MMPSense 680, Noncleavable Prosense 680 Control, Cat K 680 FAST, or VM249 (all from Perkin Elmer, Waltham, Mass) through the tail vein. Twenty-four hours after injection for Noncleavable Prosense 680 Control, Prosense 680, Prosense 750, and MMPsense 680 or 6 hours after Cat K 680 FAST and VM249, multiple sections of the tumor were removed surgically and imaged with the device. The following spectral filters (Chroma Technology, Bellows Falls, Vt.) were used: 665 ± 20 nm band-pass excitation filter, 680 nm long-pass dichroic filter, and 710 ± 12 nm band-pass emission filter for all imaging agents except Prosense 750, for which a 710 ± 32 nm band-pass excitation filter, 750 nm long-pass dichroic filter, and 810 ± 45 nm band-pass emission filter were used. Samples of muscle also were resected and imaged with the device. The pixel values of the images were normalized by the corresponding image exposure time. By using ImageJ, the normalized intensity was measured over the region of the image that contained tumor or muscle, and their ratio was calculated to obtain the tumor-to-muscle signal ratio.

### Reproduction of Intraoperative Images for Publication Display

For publication purposes, the contrast and brightness of images were adjusted so that the reader can appreciate the differences in fluorescence intensities between tumor and normal tissue. The intensity histogram of the excised tumor image was computed, and the brightness and contrast were set according to the maximum and minimum intensities of the tumor histogram. The contrast and brightness settings from the tumor image were applied to all images from the same mouse.

### Detection of Microscopic Residual Sarcoma and Intraoperative Image-Guided Surgery

Before surgery, mice with primary soft tissue sarcomas ≥200 mm^3^ were injected with 2 nmol of Cat K 680 FAST or VM249. Amputation was performed to remove grossly apparent soft tissue sarcoma. The resected tumor was imaged with the device, and pixel intensity values were normalized by exposure time to obtain a time-independent fluorescence emission rate. An intensity threshold to image residual NIR fluorescence was set based on 80% of the minimum fluorescence emission rate from the tumor image for each mouse. Then, all subsequent imaging of the animal's tumor bed was classified as either “positive” or “negative” NIR residual fluorescence based on the threshold calibration. Images obtained at the time of surgery also were analyzed after surgery to ensure correct intraoperative diagnosis using ImageJ. The intraoperative classification of residual fluorescence was based on the raw pixel counts normalized to exposure time. After surgery and intraoperative imaging of the tumor beds, the wound was closed, and mice were observed for up to 12 months for local recurrence, which was confirmed by histology. Intraoperative imaging and tumor bed assessment took approximately 1 or 2 minutes to complete. For image-guided surgery, the same imaging routine was applied, and tissue was resected until tumor beds were cleared of residual NIR fluorescence. The significance of local control according to intraoperative diagnosis was determined by Kaplan-Meier analysis.

### Yellow Fluorescent Protein Imaging

*LSL-YFP;LSL-Kras;p53^Flox/Flox^* mice with yellow fluorescent protein (YFP)-expressing sarcomas were injected with 2 nmol Prosense 680, Cat K 680 FAST, or VM249. Tumors were removed at 6 hours (VM249 and Cat K 680 FAST) or at 24 hours (Prosense 680). The resected tumor was imaged with the device, and the automatic software routine, described above, was applied. Then, subsequent imaging of the tumor bed was classified as either “positive” or “negative” residual NIR fluorescence based on threshold calibration, and the tumor bed subsequently was imaged with the imaging device with filters for YFP.

## RESULTS

### Imaging Device Design and Characterization

**Figure 1:**
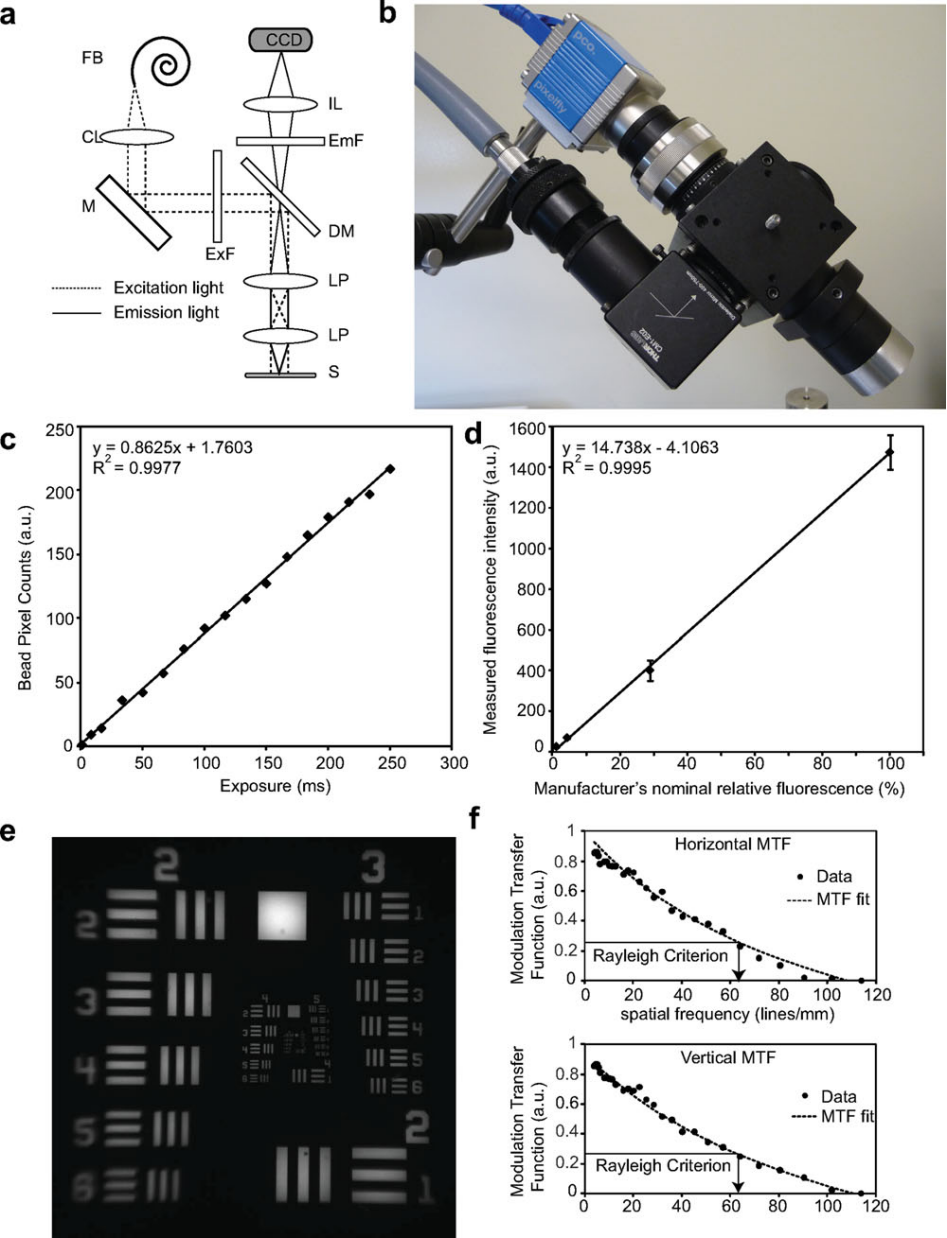
These images illustrate and characterize the prototype device. (a) Optical diagram of the intraoperative imaging device. A fiber bundle (FB) is connected to a light source and attaches to the device. A collimating lens (CL) collects the input light, and a mirror (M) reflects the light toward a band-pass excitation filter (ExF). A long-pass, dichroic mirror (DM) reflects the excitation light toward a lens-pair (LP) to illuminate the specimen (S). Fluorescence emission from the sample is transmitted through the DM and is spectrally filtered by a band-pass emission filter (EmF). An imaging lens (IL) focuses the fluorescence emission into a charge-coupled device (CCD). (b) Photograph of prototype imaging device. (c) Pixel intensities acquired from fluorescent microspheres imaged at different exposure times demonstrate a linear response of the device to exposure time (line fit). (d) Analysis indicated that the device had a linear response to 100-fold changes in intensity (line fit). Error bars indicate standard deviation from the mean. (e) Image of a standard US Air Force 1951 resolution target that was acquired with the imaging device to determine the spatial resolution of the system. (f) Horizontal and vertical contrast transfer functions are illustrated for the imaging system measured with the target image from e. The horizontal and vertical spatial frequency resolution limits are 62.8 and 63.0 cycles/mm, respectively. These correspond to approximately 16 μm of spatial resolution in both axes. MTF indicates modulation transfer function.

To detect microscopic residual cancer, we have developed a wide field-of-view imaging device for direct assessment of a tumor bed at the time of surgery. A schematic of the device's optical layout and the prototype is provided in Figure 1a,b. The device employs white light for illumination and a set of spectral filters appropriate for the fluorescent imaging agent used. Lenses are used to relay the fluorescence image at no magnification into a CCD. By maintaining a 1:1 ratio of the sample with its image, the fluorescence emissions of small numbers of cells are mapped onto multiple pixels, which improve the sensitivity and the signal-to-noise ratio of weak signals. We determined that the CCD response to exposure time and intensity was linear (Fig. 1c,d). The imaging system has a spatial resolution of approximately 16 μm according to the US Air Force 1951 calibration standard (Fig. 1e,f), and it has a relatively large field of view (9.0 × 6.6 mm). Thus, the user can scan a mouse tumor bed that measures 2 × 2 cm in a relatively short time (approximately 1 minute) with the potential of identifying areas of residual cancer as small as 16 μm.

### Tumor-to-Muscle Fluorescence Signal Ratio

**Figure 2:**
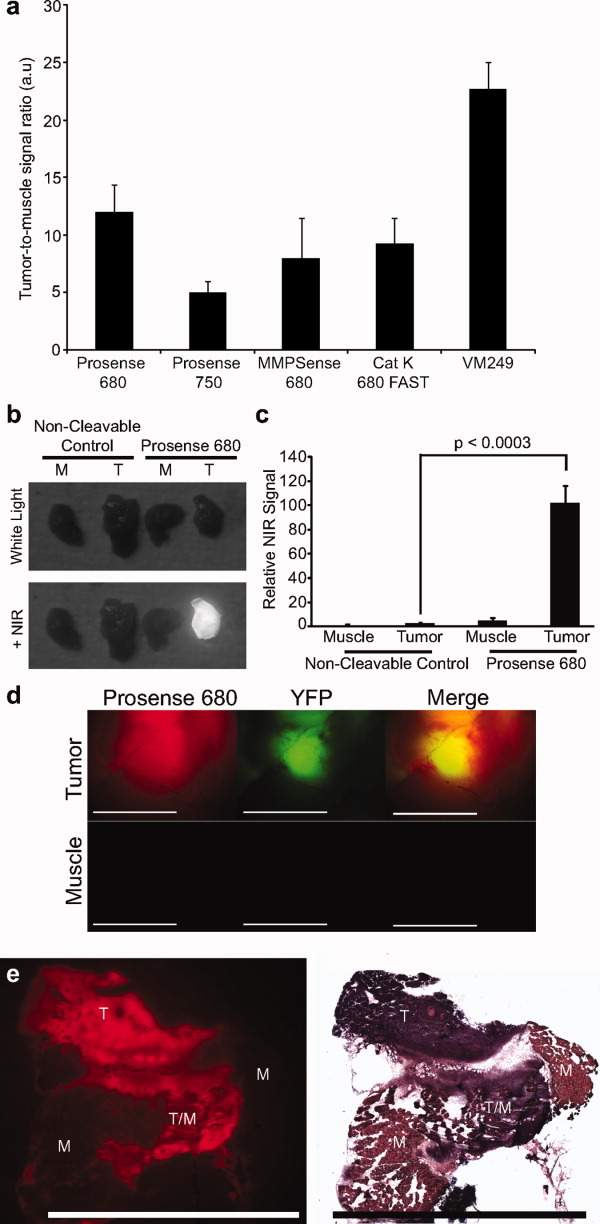
These images illustrate protease activation of near-infrared (NIR) imaging agents in autochthonous soft tissue sarcomas. (a) Fluorescence emission from sarcomas in mice injected with different probes (Prosense 680, Prosense 750, MMPSense 680, Cat K 680 FAST, and VM249) was compared with the normal in muscle from the same mouse. (b) Sarcoma-bearing mice injected with Prosense 680 or a noncleavable Prosense 680 control reveal that signal from the tumor tissue (T), but not from normal muscle (M), is observed with Prosense 680 when imaged under NIR light. (c) Fluorescence emission was quantified in sarcomas and normal muscle tissues from mice injected with Prosense 680 or a noncleavable Prosense 680 control. (d) Fluorescence imaging of a yellow fluorescent protein (YFP)-positive sarcoma after Prosense 680 injection shows that Prosense is activated in the sarcoma (tumor) but not in normal muscle from the contralateral limb. (e) Comparison of the fluorescence from Cat K 680 FAST in a frozen section with a section that was stained with H&E confirms the presence of tumor (T) in regions of high NIR fluorescence adjacent to normal muscle (M). Fluorescence imaging also detects areas of tumor invasion into normal muscle (T/M). Error bars represent standard deviation from the mean (scale bars = 5 mm).

To study intraoperative imaging, we previously developed a primary mouse model of soft tissue sarcoma in *LSL-Kras^G12D^;p53^Flox/Flox^* mice.^7^ A major challenge to detect microscopic residual cancer in vivo is to achieve sufficient contrast between the cancer cells and the surrounding normal tissue. For soft tissue sarcomas, 1 relevant adjacent structure is normal muscle. Because gene expression analysis indicates that sarcomas in the mouse model over expresses many proteases from the cathepsin family compared with normal muscle (Table 1), and studies of human sarcomas have revealed the over expression of several cathepsin proteases in soft tissue sarcomas compared with adjacent normal muscle,^13^ we used the cathepsin-activated imaging agents Prosense 680 and Prosense 750^14^ and another imaging agent activated by the matrix metalloproteinases 2 and 9, MMPsense 680.^15^ In their nominal, or “inactive,” state, these imaging agents have self-quenched fluorescence, which becomes unquenched, or “activated,” upon cleavage of the peptide backbone. Twenty-four hours after intravenous injection, we used the imaging device to measure the tumor-to-muscle fluorescence signal ratio ex vivo of sarcoma and normal muscle from the contralateral limb. The average tumor-to-muscle fluorescence signal ratio (±standard deviation) for Prosense 680, Prosense 750, and MMPSense 680 was approximately 12 ± 2.4, 5 ± 1.0, and 8 ± 3.5, respectively (Fig. 2a). The other critical, nonmalignant tissue for resection of extremity soft tissue sarcomas is the neurovascular bundle, which had a fluorescence signal similar to that observed in normal muscle (data not shown).

**Table 1:** Multiple Cathepsin Proteases Are Overexpressed in Primary Soft Tissue Sarcomas in Mice Compared With Normal Muscle

Gene	Fold Change^a^	*P*	FDR
Cathepsin A	2.9	< 1e-07	< 1e-07
Cathepsin B	2.5	3.68e-05	9.03e-05
Cathepsin C	4.3	< 1e-07	< 1e-07
Cathepsin E	2.0	.0074	.011
Cathepsin H	5.2	< 1e-07	< 1e-07
Cathepsin K	5.9	.0016	.0036
Cathepsin L	4.2	< 1e-07	< 1e-07
Cathepsin S	25	< 1e-07	< 1e-07
Cathepsin Z	4.7	< 1e-07	< 1e-07

Abbreviations: FDR, false-discovery rate.

a Fold-change represents fold-overexpression in sarcoma tissue compared with normal muscle along with corresponding *P* values and the FDR.

To determine the contribution of imaging agent activation from increased cathepsin-activity in the tumor compared with enhanced permeability and retention, we injected sarcoma-bearing mice (n = 4) with either Prosense 680 or a noncleavable Prosense 680 control and harvested tumor and muscle tissues 24 hours after injection. Significant Prosense 680 activation was apparent only in the tumor from the mouse that was injected with Prosense 680 and not in normal muscle from the same animal or in tissues from the animal that was injected with the noncleavable Prosense 680 (Fig. 2b,c). Because of the increased protease-activated signal in sarcoma compared with that in normal muscle and in the neurovascular bundle, cathepsin-activated probes may be useful for intraoperative imaging during sarcoma surgery.

### Specificity of Cathepsin-Activated Probes for Tumor Imaging

To determine whether Prosense 680 was labeling sarcomas in vivo, we used a mouse with a YFP reporter that is activated by Cre recombinase (*LSL-YFP*).^10^*LSL-YFP*;*LSL-Kras;p53^Flox/Flox^* mice that develop sarcomas after Adeno-Cre injection express YFP specifically in tumor cells. Mice with YFP-positive primary sarcomas were injected with Prosense 680 24 hours before imaging with the device. Soft tissue sarcomas had significant YFP expression, which colocalized with Prosense 680 (Fig. 2d). The NIR signal from Prosense 680 also extended beyond the YFP-expressing tumor cells, suggesting that Prosense 680 is activated by cathepsins secreted into the extracellular matrix^16^ and/or that Prosense labels nontumor parenchymal cells, such as tumor-associated macrophages.^17^ Conversely, normal muscle from the contralateral limb had no YFP expression or significant Prosense 680 activation (Fig. 2d). Furthermore, fluorescence signal from the cathepsin-activated imaging agents in frozen sections from sarcoma samples also was imaged with the device and compared with histology. The imaging agents effectively labeled microscopic tumor areas, including the invasive edge (Fig. 2e, T/M).

### Imaging Microscopic Residual Disease

**Figure 3:**
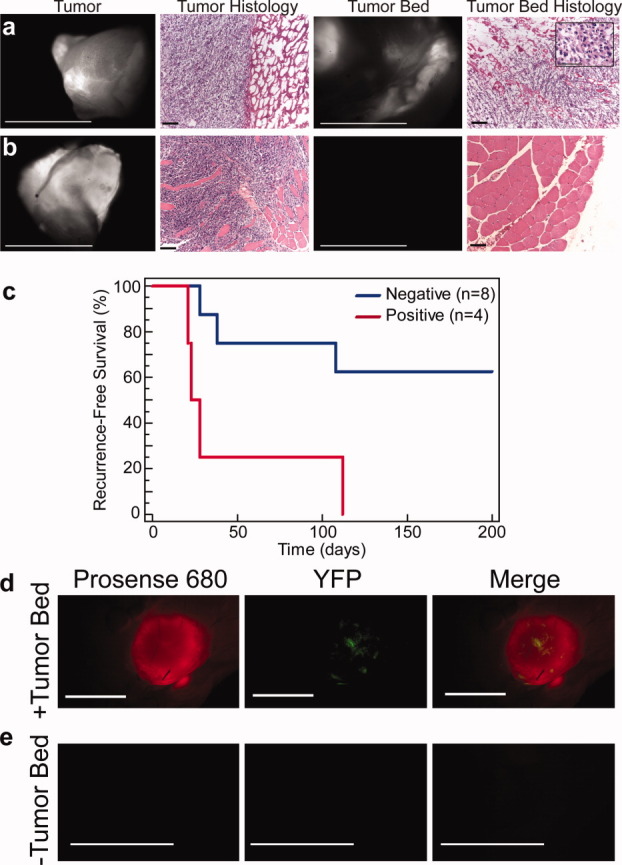
These images illustrate the correlation of residual Prosense 680 fluorescence with microscopic residual sarcoma and local recurrence. (a,b) Primary sarcomas were removed by gross total resection from mice after Prosense 680 injection, and the excised tumors (Tumor) were imaged with the device and were correlated with the histologic presence of tumor (Tumor Histology). Then, the tumor bed was imaged, and residual fluorescence suggested (a) the presence or (b) absence of residual microscopic sarcoma (Tumor Bed). H&E staining of biopsies from the tumor beds shown in a and b confirmed the presence and absence of residual sarcoma cells in the tumor bed, respectively (Tumor Bed Histology). The *inset* in the far right corner in a is a ×100 magnification of the residual sarcoma cells. (c) Imaging residual near-infrared (NIR) fluorescence in the tumor bed after injection with Prosense 680 can risk-stratify mice for local recurrence (hazard ratio, 4.7; *P* = .013). Mice with primary sarcomas that expressed yellow fluorescent protein (YFP) also were injected with Prosense 680, and the tumors were resected. (d) In tumor beds that had levels of NIR fluorescence above the threshold, YFP-positive sarcoma cells were identified in the tumor bed; whereas (e), in tumor beds without residual NIR fluorescence, there were no YFP-positive sarcoma cells (scale bars = 5 mm in a [Tumor, Tumor Bed], b [Tumor, Tumor Bed], and e; 1 mm in d; 100 μm in a [Tumor Histology, Tumor Bed Histology] and b [Tumor Histology, Tumor Bed Histology]; and 50 μm in *inset*).

We explored whether Prosense 680 could be used in conjunction with the novel imaging device to detect microscopic cancer in the tumor bed of mice during surgery. We first used an initial cohort of sarcoma-bearing mice (n = 14) injected with Prosense 680 to establish a metric capable of distinguishing tumor beds with and without microscopic residual sarcoma. For each mouse, the soft tissue sarcoma was grossly resected with a transfemoral amputation just proximal to the level of the tumor, and the excised tumor was imaged to determine its fluorescence intensity followed by imaging of the tumor bed. A biopsy of the tumor bed in the area of highest residual fluorescence was performed for histologic analysis to determine a threshold for image-based classification of the tumor bed (Fig. 3a,b). By using these data, a threshold of 80% of the minimum fluorescence emission rate of the primary tumor was established as capable of identifying mice with biopsy-proven microscopic residual disease in the tumor bed (Fig. 3a). Moreover, no mice in which the fluorescence of the tumor bed was below this threshold had biopsy-proven microscopic residual disease (Fig. 3b). When the entire cohort was followed for local recurrence, the presence of residual fluorescence above this threshold in the tumor bed predicted decreased relapse-free survival (hazard ratio [HR], 4.7; *P* = .013) (Fig. 3c).

To further validate the finding that residual fluorescence in the tumor bed correlates with microscopic residual sarcoma, we injected *LSL-YFP*;*LSL-Kras;p53^Flox/Flox^* mice (n = 5) with Adeno-Cre to generate sarcomas in which YFP was expressed only in sarcoma cells. Twenty-four hours after Prosense 680 injection, all gross tumor was removed with a transfemoral amputation just proximal to the tumor, and the tumor bed was imaged with the device. In tumor beds that had residual NIR fluorescence above the threshold, YFP-expressing tumor cells were observed in the tumor bed (Fig. 3d). In tumor beds without residual fluorescence above the threshold, no YFP-positive cells were observed (Fig. 3e).

### Intraoperative Imaging for Direct Tumor Bed Examination and Surgical Guidance

**Figure 4:**
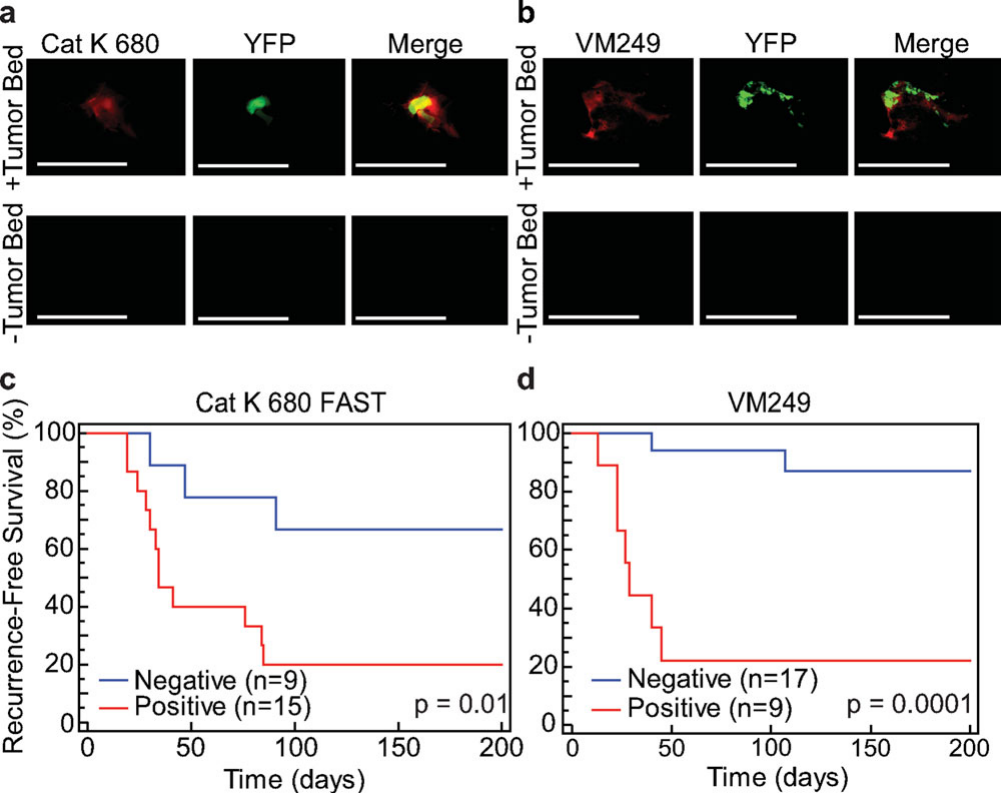
These images illustrate the correlation of residual fluorescence from the cathepsin K-specific Cat K 680 FAST probe and the multicathepsin imaging agent VM249 with microscopic residual sarcoma and local recurrence. Fluorescence imaging of mice with primary yellow fluorescent protein (YFP)-positive sarcomas injected with (a) Cat K 680 FAST or (b) VM249 reveal that the presence of residual near-infrared (NIR) fluorescence (+Tumor Bed) in the tumor bed is associated with residual YFP-positive tumor cells, whereas the absence of residual NIR fluorescence (−Tumor Bed) reveals no residual YFP-positive cells. Imaging residual fluorescence in the tumor bed after injection with (c) Cat K 680 FAST or (d) VM249 can risk-stratify mice for local recurrence (hazard ratio, 3.9 [*P* = .01] and 11.2 [*P* = .0001], respectively; scale bars = 5 mm).

Having established a threshold for residual NIR fluorescence using Prosense 680 that correlates with microscopic residual sarcoma and local recurrence, next, we tested whether residual fluorescence also correlates with residual sarcoma and local recurrence after injection with other cathepsin-specific imaging agents. Because cathepsin K is overexpressed in sarcomas (Table 1), the cathepsin K-specific Cat K 680 FAST probe was tested in addition to the multicathepsin imaging agent VM249. Cat K 680 FAST and VM249 are self-quenched imaging agents with an amino acid recognition sequence that is flanked on both sides by Vivotag S 680; however, the substrate of VM249 is susceptible to cleavage by cathepsins B, K, L, and S. These imaging agents have potential advantages over Prosense 680, including a smaller molecular weight, the ability to image tumors several hours after administration, and, for VM249, a higher tumor-to-muscle signal ratio than Prosense 680 (22.7 ± 2.3) (Fig. 2a). We injected Cat K 680 FAST or VM249 intravenously into *LSL-YFP;LSL-Kras;p53^Flox/Flox^* mice (n = 4 for each probe) with primary sarcomas that expressed YFP and performed marginal surgical resections after 6 hours. The tumor beds were imaged and classified as positive or negative for residual NIR fluorescence based on 80% of the minimum fluorescence emission rate of the primary sarcoma, as determined previously. The presence of residual fluorescence above this threshold was correlated with YFP-positive cells present in the tumor bed for both Cat K 680 FAST and VM249 (Fig. 4a,b). We also injected mice that had primary sarcomas with Cat K 680 FAST (n = 24) or VM249 (n = 26) for intraoperative imaging to determine whether residual NIR fluorescence was correlated with tumor recurrence. For these experiments, no biopsy of the tumor bed was performed to avoid removal of residual sarcoma cells that could have altered the rate of local recurrence. By using the established threshold, residual fluorescence in tumor beds after injection with Cat K 680 FAST or VM249 was correlated with tumor recurrence (Fig. 4c,d) (HR, 3.9 [*P* = .01] and 11.1 [*P* = .0001], respectively). These results demonstrate that this imaging approach can detect residual fluorescence in the tumor beds of mice after injection with an NIR imaging agent that not only correlates with microscopic residual sarcoma but that also can risk-stratify tumor beds for local recurrence.

**Figure 5:**
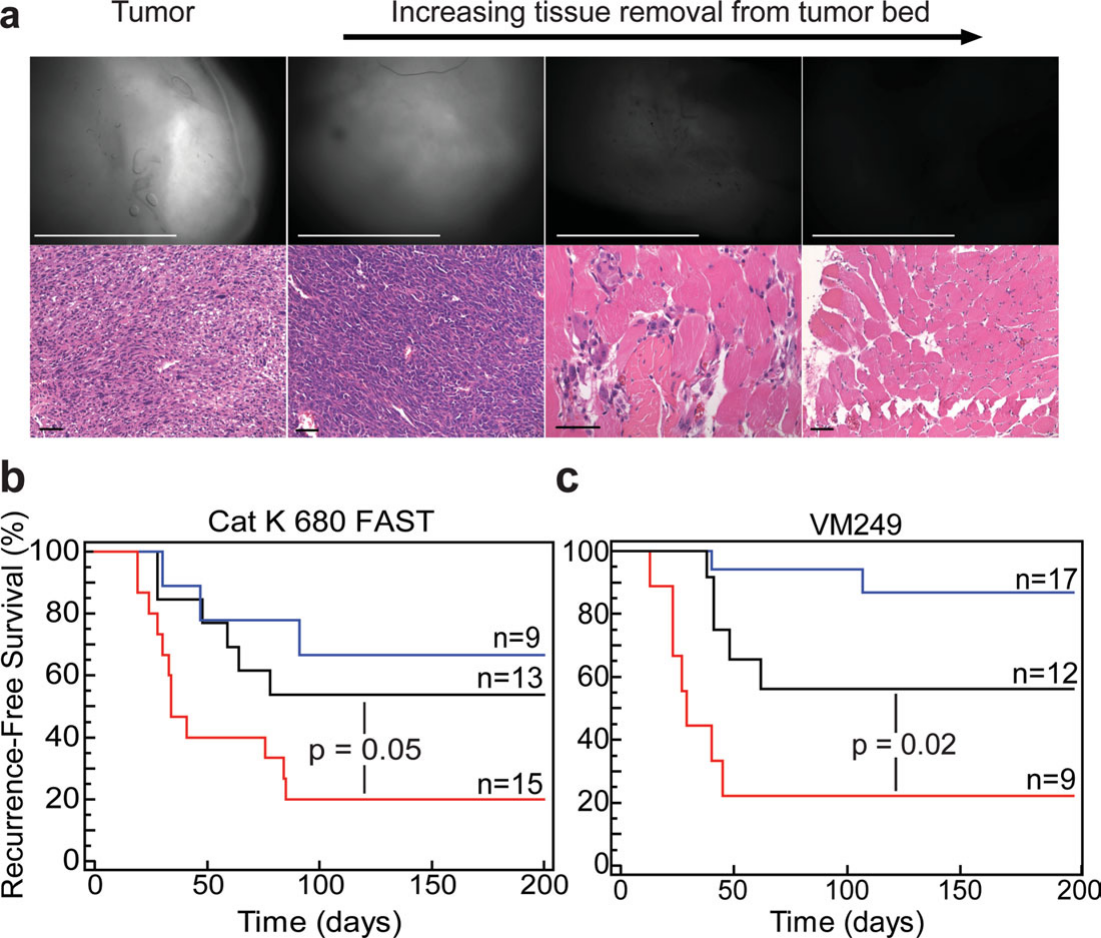
Intraoperative, image-guided surgery improves local control. (a) Representative histology is shown from the removal of tumor using the imaging device. The top row corresponds to fluorescence images of the tumor (first column) and the tumor bed after 1, 2, and 3 resections (scale bars = 5 mm). The bottom row shows the corresponding histology from a biopsy of the tumor bed at each step in the surgery (scale bars = 50 μm). The removal of residual fluorescence from the tumor bed improves local control with either (b) the cathepsin K-specific Cat K 680 FAST probe or (c) the multicathepsin imaging agent VM249 (hazard ratio, 2.5 [*P* = .05] and 3.4 [*P* = .02]., respectively). Blue lines represent single resections with no residual near-infrared (NIR) fluorescence; red lines, single resections with residual NIR fluorescence; black lines, multiple resections to a tumor bed free of residual NIR fluorescence. Numbers of mice in each cohort are noted above each line. The single-positive and single-negative cohorts are the same groups illustrated in Figure 4c,d.

A proof-of-principle experiment was designed to test the ability of the intraoperative imaging system to guide surgical resection of microscopic residual cancer from the tumor bed. Mice were injected with Cat K 680 FAST (n = 13) or VM249 (n = 12) 6 hours before surgery. An intralesional resection was performed, and the device was used to image the tumor bed and direct further surgical resection until the tumor bed was free of residual fluorescence above the established threshold. Each piece of tissue removed was collected for subsequent histologic analysis. For these mice, 2 to 4 resections were necessary to achieve a tumor bed that was free of residual fluorescence (Fig. 5a). In all cases, biopsy of the final surgical bed did not identify residual sarcoma. In mice that were injected with either Cat K 680 FAST or VM249 and underwent multiple resections, the removal of all residual fluorescence significantly improved local control (Fig. 5b,c) (HR, 2.5 [*P* = .05] and 3.4 [*P* = .02], respectively). Thus, the complete removal of residual fluorescence in 1 or more resections enhances overall local control compared with tumor beds that have residual fluorescence.

## DISCUSSION

In this study, we have demonstrated that the combination of NIR fluorescence with epi-illumination is capable of identifying microscopic residual sarcoma cells that have activated a fluorescent imaging agent in vivo. Although other groups have performed elegant experiments of image-guided surgery using transplanted tumors with different molecular imaging agents,^18^ in this study of intraoperative imaging, we used a highly invasive primary mouse model of sarcoma with the clinically relevant endpoint of local control. When the device identified tumor beds with positive residual fluorescence, this was correlated with the presence of microscopic residual sarcoma in a biopsy of the tumor bed (Fig. 3a) and direct fluorescence imaging of the tumor bed for YFP-expressing sarcoma cells (Figs. 3d, 4a,b). Moreover, the presence of residual fluorescence was correlated with local recurrence for multiple cathepsin-activated imaging agents (Figs. 3c, 4c,d) and image-guided resection of residual fluorescence improved local control (Fig. 5b,c).

By using exogenously delivered NIR imaging agents activated by cathepsin proteases, we have been able to detect microscopic residual sarcoma in the tumor bed at the time of surgery. Because cathepsin expression varies in normal tissue, these probes may not be appropriate for tumors at other sites in which the adjacent, normal tissues express high levels of cathepsin proteases, such as the liver. Our approach uses epi-illumination to deliver fluorescence excitation light and collection of fluorescence emission to generate images of the area of interest. The signal that reaches the detector will depend on the depth in tissue from which the cell emits its signal.^19^ Although we have demonstrated that our imaging device has the sensitivity to identify small clusters of tumor cells if microscopic residual cancer is at the surface of the tumor bed, it is likely that our approach will not detect a small number of tumor cells that have migrated beyond the tumor bed surface.^8^ Therefore, detection of deep, discontinuous cancer cell clusters is beyond the scope of this system. However, many cancers, including some soft tissue sarcomas, demonstrate collective invasion at the margins.^20^ Cancer cells from these tumors will migrate as sheets of cells rather than as discontinuous, single cells.^21^ We anticipate that, for many tumors, residual cancer cells after resection will be at the tumor bed surface.

Most soft tissue sarcomas in humans are treated with limb-sparing surgery involving a “wide” resection of tumor with a margin of normal tissue around the tumor in all planes. Given the relatively large tumors present in these mouse extremities, a limb-sparing approach with a wide resection of tumor and a viable residual limb was not feasible. We used amputation as a surrogate for limb-sparing surgery. Like limb-sparing resections, amputations for these tumors can result in wide, marginal, or intralesional margins based on the amputation level in relation to the proximal aspect of the tumor.

Because this technology examines the entire surgical bed in a few minutes, it can provide the surgeon with a comprehensive, real-time analysis of the tumor bed. The imaging device was purposely designed to provide the sensitivity to detect small numbers of cells after protease activation of an imaging agent. Therefore, the device does not have the resolution to identify cell-cell boundaries, because such a design would preclude the wide field-of-view, which makes this device a practical intraoperative tool that theoretically can identify areas of residual fluorescence as small as 16 μm. Recently, several molecular imaging agents have been developed that are useful for imaging cancer. These agents target over expressed proteolytic enzyme^14^, ^15^ and surface markers^22^, ^23^ to provide high contrast between the fluorescence emission of cancer and normal tissue. To improve specificity and enhance tumor contrast, it may be necessary to use a cocktail of these imaging agents that target tumor cells. Such a cocktail could be tailored to an individual patient by examining the expression of target molecules from a biopsy sample before surgery. Despite the significant time and expense required to develop NIR imaging agents for clinical use, the availability of NIR imaging agents, such as those activated by cathepsin proteases, would greatly improve the outlook for translating intraoperative molecular imaging to the clinic. Successful translation of intraoperative NIR imaging into the clinic has the potential to identify patients who have microscopic residual sarcoma, which could be used to direct adjuvant therapy.

In summary, we have combined a wide field-of-view imaging device with a series of NIR cathepsin protease-activated imaging probes to detect microscopic residual sarcoma in mice in vivo. We observed that the presence of residual NIR fluorescence can risk-stratify animals for local recurrence, and we demonstrated the feasibility of using this approach for intraoperative image-guided surgery to direct additional surgical resection that removes residual sarcoma and improves outcomes. To our knowledge, this is the first time that a primary tumor model system, in the absence of tumor transplantation, has been used for intraoperative imaging studies with local control as the endpoint. These results demonstrate the power of molecular imaging for intraoperative visualization and provide a preclinical platform for testing new imaging agents and systems.

## FUNDING SOURCES

This work was supported by a Damon Runyon-Rachleff Innovation award (to D.G.K.), a joint American Cancer Society-Canary Foundation Postdoctoral Fellowship (to R.D.D.), and a Marble Foundation Grant awarded to the Massachusetts Institute of Technology Koch Institute for Integrative Cancer Research.

**CONFLICT OF INTEREST DISCLOSURES**

W.D.L. is a cofounder and interim chief executive officer of Lumicell Diagnostics, Inc., a company that commercializes in vivo imaging systems. M.G.B. is a cofounder of Lumicell Diagnostics, Inc.; L.G.G. and D.G.K. are members of the Scientific Advisory Board of Lumicell Diagnostics, Inc.; and J.M.F. is a consultant for Lumicell Diagnostics, Inc.
